# 

**Published:** 1921-04-23

**Authors:** 


April 23, 1921] [Price Twopence

### The Hospital

The Workers' Newspaper of Administrative Medicine and Institutional Life, Administration, National Insurance and Health.

### CONTENTS

**King Edward's Fund Policy** ... ... ... ... 57
**Consult the Doctor** ... ... ... ... ... 57
**The Almoners' Council and Patients' Payments** 58
**Annual Meetings of Hospitals** ... ... ... ... 58
**Notes of the Week** ... ... ... ... ... 59
Hospital for Sick Children—Bradford Again—Large Gift to the West London Hospital—The Wellhouse Muddle—A Competition for Architects—The Nichols Prize—Ward Let as a Flat—A Suggestion to Manchester—Charities Competing with Industry—The Cost of In-patients—Parish Funds for Hospitals—A Southampton Success—A Medical Officer's Motor-car—The First Fruits of a Bold Plan.
**The Custom of Drinking Tea** ... ... ... ... 61
Facts For and Against.
**The Short Cut to Sanity** ... ... ... ... 62
**Hospital Engineering Reforms** ... ... ... 63
Possible Economies with Steam Boilers.
**The Health of our Eyes** .. ... ... ... ... 64
Some Notes on an Everyday Problem.

### CONTENTS

**The Public Well-Being** ... ... ... ... ... 65
Instructing the Public—Medical Officers and Food Problems—Waist-bands for Fat Men.
**The Preservation of the Voluntary System** ... 67
Interim Report by King's Fund Policy Committee.
**The Editor's Letter-Box** .. ... ... ... ... 68
The Practice of Birth-Control—Superannuation and the Young Nurse.
**The Matrons' and Sisters' Department** ... ... 69
The Pay of the Nurse.—VII. Causes of Low Salaries. Security for the Masseur and the Masseuse.
**Round the Hospitals** ... ... ... ... ... 70
**Nurse Registration in Ireland** .. ... ... ... 71
Conditions of Admission to the Register.
**The Organised Practice of Massage** ... ... ... 72
**Nurses Want a Career** ... ... ... ... ... 73
II.—The Absence of Educational Grades.
**The College of Nursing (Limited by Guarantee)** 74
What the Local Centres are Doing.

